# Child psychosis-risk screening system diagnostic specificity: differentiation of schizophrenia spectrum and neurodevelopmental disorders

**DOI:** 10.1192/j.eurpsy.2023.630

**Published:** 2023-07-19

**Authors:** Y. Hamasaki, Y. Sakaue, M. Matsuo, R. Sanada, T. Nakayama, S. Michikoshi, S. Ueba, T. Hikida

**Affiliations:** ^1^Kyoto Women’s Universty, Kyoto; ^2^Department of Pediatrics; ^3^Department of Psychiatry, Shiga University of Medical Science, Shiga; ^4^Kyoto Women’s University, Kyoto; ^5^Saiseikai Moriyama Municipal Hospital, Shiga; ^6^Osaka University, Osaka, Japan

## Abstract

**Introduction:**

Adolescents presenting with a first psychotic episode often have a long history of pediatric treatment. However, there is insufficient evidence of children’s subclinical characteristics in non-psychiatric settings. To address this issue, we retrospectively studied schizophrenia spectrum disorder (SSD) patients to identify characteristic patterns of subclinical psychological, behavioral, and physical problems in childhood. In the previous study, we had developed the child psychosis-risk screening system (CPSS) that incorporates this pattern as a risk evaluation algorithm (Hamasaki et al. BMC Psychiatry 2021; 21, 57).

**Objectives:**

In this present cross-sectional study, we evaluated the specificity of the CPSS to identify the risk of psychosis in pediatric and psychiatric patients and determine its discriminatory power and cutoff values.

**Methods:**

To identify the risk of developing psychosis in pediatric and psychiatric outpatients, we evaluated data from 336 patients aged 6–18 years selected for the present study using the CPSS. We defined six major diagnostic categories i.e., Neurodevelopmental Disorders, SSD, Depressive Disorders, Anxiety Disorders (including Obsessive-Compulsive Disorder), Somatic Symptom Disorders, and Others to examine the specificity of the CPSS variance in diagnosis. We analyzed the receiver operating characteristic (ROC) curve using the onset of schizophrenia spectrum as the outcome and determined the discriminatory power and cutoff values of CPSS.

**Results:**

We found significant differences in CPSS variance among the diagnostic categories (Kruskal–Wallis test; p<0.001), especially between SSD and neurodevelopmental disorders (Bonferroni method; p=0.001). Similarly, significant differences were identified in variance when comparing the CPSS for each neurodevelopmental disorder category and SSD, particularly between SSD and attention deficit hyperactivity disorder (ADHD) (Bonferroni method; p<0.001) and SSD and autism spectrum disorder (ASD) (Bonferroni method; p=0.004). CPSS showed sufficient discriminatory power for SSD diagnosis (area under the ROC curve=0.853 [95% confidence interval: 0.774–0.931]). The cutoff value for the risk of SSD was determined to be 3.94, achieving the best mean of the sum of sensitivity (90.9%) and specificity (84.0%). 18.3% of patients (12.5% pediatric and 29.1% psychiatric) were identified as risk groups above the cutoff value.

**Conclusions:**

These results suggest that CPSS can be applied in pediatric clinical practice not only for early detection and risk identification of psychosis but also for differentiation from neurodevelopmental disorders. If early identification of psychosis risk in pediatrics becomes possible, discussions regarding effective prevention during the critical period of psychosis will become increasingly important.

**Disclosure of Interest:**

None Declared

